# Recruitment to doping and help-seeking behavior of eight female AAS users

**DOI:** 10.1186/s13011-016-0056-3

**Published:** 2016-03-05

**Authors:** Annica Börjesson, Nina Gårevik, Marja-Liisa Dahl, Anders Rane, Lena Ekström

**Affiliations:** Department of Laboratory Medicine, Karolinska Institutet, Karolinska University Hospital, SE-141 86 Stockholm, Sweden; Department of Clinical Pharmacology, Karolinska University Hospital, SE-141 86 Stockholm, Sweden

**Keywords:** Doping, Women, Anabolic androgenic steroids, Clenbuterol

## Abstract

**Background:**

Doping with anabolic androgenic steroids in sports has now developed to a widespread use of these agents among young people outside the sport. This is of major concern to the society. The purpose of the use is mainly for aesthetic reasons and is seen as a male phenomenon. But use also occurs in women where the knowledge is scarce. Our aim was to identify the pattern of doping agents in eight female cases and compare them with similar data from men.

**Methods:**

Eight female users were recruited through Anti-Doping Hot-Line, a national telephone counseling service on doping issues during the years 1998–2004. The use was confirmed with urine doping analysis at the Doping Laboratory. The characteristic of use, co-use of narcotics/other doping agents, exercise pattern, adverse-side effects, family history and reason to begin was evaluated.

**Results:**

The women used on average 1.9 different anabolic androgenic steroids and clenbuterol preparations. Ephedrine and growth hormone were co-used in five and one of the women, respectively. Three women reported co-use of narcotics (cannabis and cocaine).

The average duration of anabolic agent use before contacting health care was 58 weeks (range 7–104). Side effects for anabolic androgenic steroids (*n* = 5) included voice changes, clitoral enlargement, body hair growth, whereas women using clenbuterol (*n* = 2) reported tachycardia and depression. All women except one had a man in close relationship encouraging them to begin with the doping agents.

**Conclusions:**

The use of doping agents in our eight women was different from that in male users. The women used less doping agents and were more prone to contact the health care, at an earlier stage, probably due to the adverse effects. The co-use with ephedrine, growth hormone and cannabis appeared to be in the same range as in men. This is the first study showing that a man in close relationship may motivate a woman to use anabolic agents.

## Background

Anabolic androgenic steroids (AAS) including testosterone, other endogenous androgenic hormones and synthetic compounds structurally related to these compounds, are commonly used by athletes to improve muscle mass and enhance exercise performance. AAS are the most frequently detected doping agents, testosterone being the predominant steroid (http://www.wada-ama.org). Notably, the use of these agents among nonprofessional athletes, as well as among individuals who want to enhance their physical appearance, is a growing public health problem and has become a major concern to the society [1–3].

Even though AAS use is mainly considered a male phenomenon, it is not limited to men. It has been estimated that the lifetime prevalence of AAS use is around 0.1 % in women [4]. Questionnaire surveys indicate that the numbers may be even higher among adolescent girls where lifetime prevalence values of 0.1–7.3 % have been reported [4–8]. However, some of these studies have probably over-estimated the prevalence since the questions used sometimes failed to distinguish between anabolic steroids, corticosteroids, and over-the-counter supplements that the respondents might have confused with steroids [4]. Nevertheless, AAS may develop to a health problem also in women [7] and preventive measures in the society are warranted [9].

Even though the illicit use of AAS may be considered a health problem in women, little is known about this group of users. The use of AAS in women and adolescent girls is highly associated with sport activity, body-building and weightlifting [7, 10], but the motives for starting the use of AAS have not been investigated in women.

In general, women appear to prefer doping class agents other than AAS, particularly those associated with weight loss, such as ephedrine and clenbuterol [7]. Clenbuterol which is a long-acting beta-2-agonist used in veterinary medicine [11] is classified as an anabolic agent according to WADA (www.wada-ama.org), since it exerts anabolic effects [12].

The negative side effects of AAS use in women include enlargement of the clitoris, deepening of the voice, increased facial hair, menstrual disorders, and acne. Changes in mood such as depression, irritation and aggression have also been reported [5, 7, 13, 14]. To our knowledge, the negative effects of clenbuterol abuse in women have not been described in literature.

Our aim was to identify the pattern of AAS and clenbuterol use in women and compare with similar data reported in men. The causes for initiating an illicit use, co-use of other doping agents and narcotics, as well as any side effects, reported or observed, were also assessed.

## Methods

**Fig. 1:**
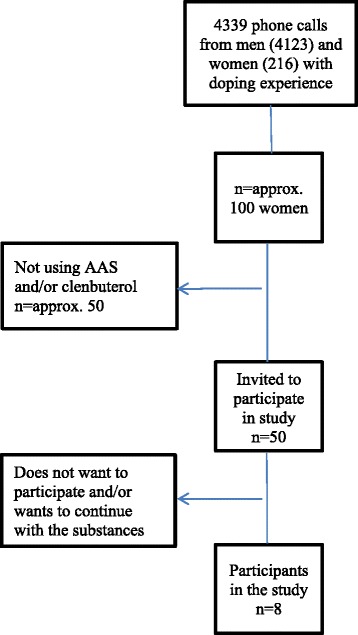
Flow chart of the participant recruitment through the study period 1998–2004

Eight women were recruited to the study between 1998 and 2004 via the Anti-Doping Hot-Line at the Dept of Clinical Pharmacology, Karolinska University Hospital, a free telephone counseling service for individuals affected by, or concerned with use of doping agents [2]. During the study period the Anti-Doping Hot-Line received 4339 phone calls and of these, 216 phone calls were from women with own doping experience [2]. Some of the women were calling several times and therefore the exact numbers of women calling during this period was not documented. Approximately 50 women were concerned about their side-effects of AAS and/or clenbuterol and were asked to participate in this study. A genuine desire to give up using anabolic agents (AAS or clenbuterol) was a prerequisite to be included. A flow chart describing the collection procedure is presented in Fig. 1.

Participation was commenced after informed consent, and no economical remuneration was given. Male users were also recruited (*n* = 56) and the results regarding their demographics, side effects and co-abuse have already been published [15, 16]. The project was approved by the Ethics Committee of the Karolinska Institutet, Stockholm, Sweden. Blood and urine samples were collected at the visit. The urine samples were analyzed for AAS and other doping agents using the methods employed at that time in the WADA accredited Doping Laboratory, and for narcotic substances by routine screening methods at the Drugs of Abuse Laboratory, both at the Department of Clinical Pharmacology, Karolinska University Hospital. The cholesterol profile (HDL, LDL) and hemoglobin (Hb) were analyzed by routine methods at the Department of clinical chemistry (Karolinska University Hospital). All participants were personally interviewed by a study nurse including questions about 1) demographics 2) details about AAS use history and pattern 3) motives for starting AAS 4) co-use of other doping agents and narcotic substances 5) exercise pattern 6) family background and 7) any experienced side effects. If necessary, individuals were referred to qualified medical specialist at the hospital clinics of psychiatry or endocrinology and/or to a gynecologist.

## Results and discussion

### Background information and motives for starting AAS use

Most of the women reported a troublesome family history such as growing up without parents (*n* = 2), split families (*n* = 5), parents that were violent (*n* = 1), mentally ill (*n* = 1) or alcoholics (*n* = 1). One woman reported a suicide attempt and eating disorder (anorexia and bulimia) during her youth. In a previous study, Ip et al. noted that 25 % of female AAS users display bulimia, whereas eating disorders in male AAS users are very rare [5]. No one reported any sexual abuse or harassments which have been reported in other studies of female AAS users [5, 17, 18].

In agreement with previous studies most of the females herein (*n* = 7) were involved in bodybuilding and/or strength training [5, 7, 10] and four of them were competing in body-building (Table 1). K7 was the only woman that did not report any involvement in strength training, but had interest and participated in sport activities including handball.

**Table 1:** Self-reported motives for using AAS/clenbuterol in eight female users of AAS

Subject	Athletics	Motives for starting AAS
K1	Strength training/competition in bodybuilding	Wants to increase performance. Lives with AAS using boyfriend
K2	Strength training/competition in bodybuilding	Her boyfriend uses AAS and insisted her to take clenbuterol even though she did not want to. Planning to use AAS
K3	Strength training	To increase muscle size and decrease fat
K4	Strength training	To increase performance, Convinced by AAS using boyfriend
K5	Strength training, Group training, Works in a gym	Recommendation from her ex-partner which was a AAS user
K6	Playing handboll and other sports	Have been tricked by her father who used AAS to take clenbuterol for weight loss
K7	Strength training/competition in bodybuilding	Convinced by AAS using boyfriend
K8	Strength training/competition in bodybuilding	Convinced by AAS using boyfriend

Of the eight women included in the study, six reported that their boyfriend was using AAS, and that the boyfriend impact on the decision to start had been crucial. One woman (K6) was deceived by her father to take clenbuterol for losing weight, and she believed that, based on her observed effects that her father also gave her AAS. Another woman (K3) was the only participant that took the decision on her own to start the use of an anabolic agent without being deceived or influenced by a man, (Table 1).

Our findings are interesting as, to our knowledge, there is only one previous case report of a woman who was introduced to use AAS by her boyfriend [17]. This novel finding that AAS/clenbuterol use in women is initiated by a man in a close relationship, would be interesting to investigate further in other AAS using female populations. AAS use in men is predominantly motivated by a desire for their anabolic effect, i.e., to attain a nice and strong body [19–22]. The influence of a peer seems to be rare among male AAS users although such cases have been reported [20, 23].

### Characteristics of the female AAS-users

The mean age was 23.0 years (SD 5.7, range 16–31) and the participants reported to have started their AAS and/or clenbuterol use at the mean age of 21.7 years (SD 5.9, range 15–29). The latter is similar to that reported in men included in the same study population [15], as well as among AAS using men seeking help at a substance abuse center [19].

The duration of AAS/clenbuterol use when contacting the Anti-Doping Hot-Line varied between 7 weeks and 2 years (mean 58 weeks). At the visit the subjects reported that their latest intake was between 0 and 25 weeks earlier (Table 2). In men the reported mean duration of AAS when contacting the Anti-Doping Hot-Line was 5.2 (0.5–17) years. [15] Similar figures (0.5–17 years) have also been reported elsewhere [19]. Thus, females reported markedly shorter duration of anabolic agents use than men, which could be explained by negative side effects being more bothering or apparent, and causing more concerns in women, resulting in women to be more prone to contact health–care givers. In general unwanted AAS related side effects are more often reported in women than in men [5], probably since women have lower testosterone levels and are more sensitive to exogenous administration of steroidal agents [24].

**Table 2:** Characteristics of AAS use in the study group at the time of inclusion

Subject	Age	Self- Reported, AAS/clenbuterol	Identified in urine analysis	Last Intake (weeks)	Duration of AAS use	Other doping agents used	Reported Narcotics	Urine analysis
K1	24	Primobolan, Winstrol	methenolone (1621 ng/mL) stanozolol (116 ng/mL)	3	2 years		Cannabis	Cannabis
K2	27	Clenbuterol	clenbuterol (42 ng/mL)	0^a^	8 months		None	Negative
K3	29	Clenbuterol	Negative	1	3.5 months	ephedrine	None	Negative
K4	31	Winstrol, Primobolan	stanozolol (56.8 ng/mL) nandrolone (17.4 ng/mL)	14	2 years	Growth hormone, ephedrine	None	Negative
K5	20	Winstrol	Negative	25	7 weeks		Cocaine	Negative
K6	16	Clenbuterol	Negative	12	3 years	ephedrine	None	Negative
K7	21	Metandrostenolone, Deca-Durabol, Clenbuterol	Negative	1	2 years	ephedrine	None	Negative
K8	16	Deca-Durabol, Stanozolol, clenbuterol	nandrolone (4947 ng/mL) stanozolol (84 ng/mL)	5	1 year	ephedrine	Cannabis	Cannabis

^a^she used the day before visit

### AAS/clenbuterol use (self-reported and confirmed by analysis)

Of the eight women included in the study, four reported use of clenbuterol, four reported use of Winstrol (stanozolol), two individuals use of Primobolan (methenolone enanthate) and two Deca-Durabol (nandrolone). One of the women claimed that she had probably been using an unknown AAS previously. Of the eight women, three were positive for all the doping agents that they actually reported (Table 2). The fact that the self-reported compounds did not fully match the substances found in urine tests is consistent with previous studies in women [7] and in men. [25] One of the women tested positive for nandrolone, a substance she did not report. This could be due to nandrolone contamination of any other agents she used, including nutritional supplements, as it has been reported that nandrolone has been found in supplements [26, 27]. It could also be due to memory default, in particular since nandrolone can be detected for several months after the last intake [15, 28].

The females used on average 1.9 (range 1–3) different AAS/clenbuterol preparations. The women that were only using AAS, reported on average 1.6 (range 1–2) different AAS compounds. This is similar to the results of an internet based survey showing that female AAS users used on average 1.2 (range 1–2) AAS per cycle, with the corresponding number in men being higher 2.3 (range 1–7) [5].

The supposed basis for using several types of AAS is to maximize androgen receptor binding and to activate multiple steroid receptor sites. Only two of the women reported use of an injectable steroid. In contrast, the majority of males use injectable AAS formulations [29, 30]. This gender difference with females preferring lower doses and oral administration has also been acknowledged by Ip et al. [5] Women are probably using lower doses and fewer AAS agents to minimize the negative side effects of AAS.

### Co-use of other substances

In addition to AAS/clenbuterol, ephedrine and growth hormone were co-used in five and one of the women, respectively. These doping agents are known to be more common among AAS users as compared to women not using AAS [7, 31]. According to a metaanalysis conducted by Sagoe et al. [32] both ephedrine and growth hormone are on the 10-top list as the most popular substances to co-use in males using AAS. Three women reported co-use of narcotics (cocaine and cannabis), and two of them tested positive for cannabis. This is a somewhat higher proportion than observed in a previous study where 24 % of female AAS users were reported to use narcotics [18]. The corresponding number in men included in the male population collected at the same time period, was only 5 % tested positive on narcotics whereas about 40 % reported a co-use [15]. Similar frequencies were also reported in more recent studies where 30–50 % of AAS using males were also using cannabis [20, 22, 33, 34]. These results indicate that the prevalence of narcotic use, at least of cannabis, may be similar in AAS using women and men.

### Reported side effects

All AAS using women reported the classical side effects associated with AAS in women such as clitoral enlargement and voice changes. Other side effects were acne, body hair growth, menstrual disturbance and mood changes i.e., irritation, aggression and depression (Table 3). There are indications that women have a tendency to underestimate their side effects. It has been noticed that even though female AAS users exhibit apparent deep voice, many do not report that as a side effect [7]. It is possible that since the women included in our study had spontaneously contacted the anti-doping hotline, they were more alerted and concerned about their side effects than of female AAS users in general.

**Table 3:** Adverse effects, reported and observed, in women using AAS or clenbuterol

Subject	Self-reported adverse effect	Cholesterol mmol/L	HDL/LDL mmol/L	HB g/L
K1	Clitoral enlargement, voice change, body hair growth, menstrual disorders	4.2	0.7/3.3	135
K2	Tachycardia, depression	3.2	1.2/1.6	125
K3	Tachycardia, depression, mood changes, edema	5.9	1.4/4	NA
K4	Clitoral enlargement, voice change, menstrual disorders, acne, body hair growth	3.8	0.6/3	154
K5	Enlarged clitoris, voice change, body hair growth, acne, menstrual disorder	3.6	1.5/1.9	134
K6	Clitoral enlargement, voice change, menstrual disorders, body hair growth, aggressiveness, acne, memory disturbance, stretch marks, tremor, tachycardia, depression	3.9	1.2/2.1	134
K7	Clitoral enlargement, voice change, menstrual disorders, acne, mood changes, depression, lost hair head	4.3	1.9/2.0	134
K8	Clitoral enlargement, voice change, mood swings, stretch marks, reduced breast	3.2	0.7/2.2	128

The two women on clenbuterol reported only depression and tachycardia (Table 3). Case studies indicate that clenbuterol may cause cardiac ischemia and rhythm disturbances [35] and myocardial infarction [36] in body-building men. However, data on illicit use of clenbuterol in women are scarce. Patients reporting clenbuterol overdose also present with tachycardia as well as tremor, chest pain, headache, nausea etc. [11]. One of the women using only clenbuterol also reported tremor as a side effect, which is a common side effect of all beta-adrenoceptor agonists. In fact, some athletes discontinue the administration of clenbuterol due to disturbing tremor [37]. Clenbuterol is promoted as an anabolic agent with fat burning, weight loosing and performance enhancing properties [35, 38]. Since it is a popular doping substance among AAS using women [18] and men [39] as well among elite athletes (https://www.wada-ama.org) it is important to understand the medical consequences of using this drug.

### Cardiovascular biomarkers

It is well known that supra-physiological doses of AAS have detrimental effects on the lipid profile and Hb levels in men as reviewed in [40], whereas studies in women are scarce. The mean (±SD) concentrations of total cholesterol, HDL, LDL and Hb were 4.0 ± 0.9 mmol/L, 1.2 ± 0.5mmol/L, 2.6 ± 0.9 mmol/L and 135 ± 10 g/L, respectively (individual data given in Table 3). These values are in the normal range for women and were not as high as in male AAS users where mean LDL and Hb values of 3.2 mmol/L and 162 g/L, respectively, were observed [15, 16]. However, it is not possible to draw any conclusions based on this low number of individuals since the lipid and Hb profile is highly dependent not only on dose and type of AAS but also on age, diet, exercise, genetics etc. [41–46].

Our report has some limitations that need to be addressed. First, the study is only based on 8 cases, a much smaller number than many studies in men. However, studies in women are more difficult since AAS use is much rarer. Second, the way of recruitment implicates a selection bias since the women contacted the health care on own initiative. Moreover, our selection criteria did not allow us to conduct studies in women that did not want to quit their AAS/clenbuterol use. It is possible that these women did not experience side effects (at least not yet) and that the utilization profile was different. For these reasons our results may not be representative for other groups of AAS and/or clenbuterol using women. However, because of the scarcity of knowledge on doping in women we believe the results presented here are of interest. One advantage of this study is that it was not based solely on self-reported data, since AAS intake was confirmed by urine analyses and the nurse’s assessment of the health status.

## Conclusion

This is the first study showing that a male partner or another man in close relationship may trigger use of AAS/clenbuterol in women. The women used fewer AAS substances and they were more prone to contact the health care at an earlier stage than men. This may be explained by a higher female inclination to experience many of the side effects. In accordance with earlier studies, several adverse effects were noted including clitoral enlargement, voice changes, menstrual disorders, acne, and mood changes. Ephedrine, growth hormone and cannabis were substances that were frequently co-used. These substances are also popular among AAS using males. General knowledge about AAS use in sports and in the society is important for the medical community and other people dealing with AAS users. This will improve our strategies for prevention and treatment. Our results indicate that not only treatment directions but also future preventing programs may be different between men and women.

### Ethics, consent and permissions

This study was reviewed and approved by the Ethics Committee of the Karolinska Institutet, Stockholm, Sweden (Dnr: 186/98). Consent to publish from the participants in this study has been given.
